# Characteristics of Normal-tension Glaucoma Patients with Temporal Retinal Nerve Fibre Defects

**DOI:** 10.1038/s41598-020-63486-7

**Published:** 2020-04-14

**Authors:** Hae Ri Yum, Hae-Young Lopilly Park, Chan Kee Park

**Affiliations:** 1 Department of Ophthalmology, Eunpyeong St. Mary’s Hospital, College of Medicine, The Catholic University of Korea, Seoul, South Korea; 2 Department of Ophthalmology & Visual Science, Seoul St. Mary’s Hospital, College of Medicine, The Catholic University of Korea, Seoul, South Korea

**Keywords:** Glaucoma, Eye manifestations

## Abstract

Glaucomatous visual field (VF) damage usually involves in the Bjerrum area, which refers to outside the central 10° region. However, some reports suggest that structural damage to the macula occurs even in the early stages of glaucoma. We investigated the characteristics of normal tension glaucoma (NTG) patients with temporal retinal nerve fibre layer (RNFL) defects. Ninety eyes from 90 subjects including 30 normal eyes, 30 eyes of 30 patients with normal-tension glaucoma with temporal RNFL defects, and 30 eyes of 30 patients with normal-tension glaucoma with inferotemporal or superotemporal RNFL defects were enrolled. The best-corrected visual acuity (BCVA) decreased significantly in glaucomatous eyes with temporal RNFL defects as compared with in controls and glaucomatous eyes with inferotemporal or superotemporal RNFL defects. VF tests showed more frequent central or cecocentral VF defects involving the central 10° region in glaucomatous eyes with temporal RNFL defects. VF defects were more frequently detected on short-wavelength automated perimetry (SWAP). Eyes with temporal RNFL defects had generally reduced ganglion cell-inner plexiform layer (GCIPL) thickness. In addition, the BCVA, GCIPL thicknesses, and SWAP findings were significantly different in glaucoma patients with temporal RNFL defects according to their colour vision deficiency, not RNFL thickness or standard automated perimetry (SAP) results.

## Introduction

Glaucoma is a major cause of blindness worldwide^1^. It is a neurodegenerative disorder characterized by a progressive loss of retinal ganglion cells (RGCs) and their axons. Retinal nerve fibre layer (RNFL) defects in glaucoma patients are most frequently found in the inferotemporal region, followed by the superotemporal region^2,3^. Visual field defects caused by glaucoma usually involve the Bjerrum region, which refers to the arcuate-shaped scotoma outside the central 10° region^4^. However, some glaucoma patients have RNFL damage close to the central region in the retina, the temporal region of the optic disc, and present with paracentral or central scotoma in the visual field^2^. Several studies have reported that early glaucomatous damage can affect the macular area^5–10^. A recent investigation demonstrated that macular involvement and central and paracentral VF defects can occur even in the early stages of glaucoma^11,12^. In addition, eyes with initial parafoveal scotoma have significantly different baseline characteristics from eyes with peripheral scotoma in glaucoma^13^.

We assumed that normal-tension glaucoma (NTG) patients with temporal RNFL defects may have apparent structural differences regarding optic nerve head (ONH) and visual field defects as compared with NTG patients with inferotemporal or superotemporal RNFL defects.

Therefore, we investigated the characteristics of NTG patients with temporal RNFL defects. An analysis was conducted to compare optic disc parameters and the RNFL thickness profile with macular ganglion cell analysis (GCA) maps determined by Cirrus high-definition optical coherence tomography (HD-OCT) and various visual field tests among normal controls, NTG patients with inferotemporal or superotemporal RNFL defects, and NTG patients with temporal RNFL defects.

## Results

This study included 30 healthy eyes from 30 healthy control subjects, 30 eyes from NTG patients with temporal RNFL defects, and 30 eyes from NTG patients with inferotemporal or superotemporal RNFL defects. The intraocular pressure (IOP) values of eyes with temporal RNFL defects were similar to those of eyes with inferotemporal or superotemporal RNFL defects. Table 1 lists the other baseline characteristics of the three groups. There were no significant differences in age, sex, spherical equivalent, axial length, central corneal thickness, or IOP between the groups. In addition, the systemic conditions such as diabetes mellitus, hypertension, and vascular events showed no significant differences between the groups. However, the best-corrected visual acuity (BCVA) was worse and more frequent colour vision deficiencies were found in the temporal RNFL defects group as compared with in the control and inferotemporal or superotemporal RNFL defects groups. There were no significant differences in disc-fovea angle or vertical/horizontal optic disc rotation among the three groups. The optic disc rotations did not affect the OCT parameters and RNFL distribution.

**Table 1:** Comparison of the clinical characteristics of glaucoma patients with temporal RNFL defects with those of the controls and glaucoma patients with inferotemporal or superotemporal RNFL defects.

	Control (n = 30)	Glaucoma withsupero/inferotemporal RNFL defect (n = 30)	Glaucoma with temporal RNFL defect (n = 30)	P-value^a^	P-value^b^
Age, years	49.87 ± 13.76	52.07 ± 13.67	53.93 ± 14.49	0.269*	0.610*
Sex, male:female	13:17	17:13	11:19	0.598^†^	0.121^†^
Family history, n (%)	0 (0)	3 (10)	7 (23.3)	0.237^†^	0.299^†^
Diabetes mellitus, n (%)	2 (6.7)	3 (10)	2 (6.7)	1.000^†^	1.000^†^
Systemic hypertension, n (%)	4 (13.3)	7 (23.3)	9 (30)	0.506^†^	0.771^†^
Diastolic blood pressure, mmHg	75.16 ± 9.39	78.00 ± 12.07	77.06 ± 12.32	0.868*	0.964*
Systolic blood pressure, mmHg	123.32 ± 17.53	118.24 ± 15.65	125.72 ± 15.63	0.904*	0.366*
**History of vascular events, n (%)**					
Migraine	5 (16.7)	6 (20)	5 (16.7)	1.000^†^	1.000^†^
Cold extremities	4 (13.3)	4 (13.3)	3 (10)	1.000^†^	1.000^†^
Orthostatic hypotension	0 (5)	0 (0)	1 (3.3)	1.000^†^	1.000^†^
Spherical equivalent, diopter	−0.36 ± 1.05	−2.15 ± 2.86	−1.06 ± 2.80	0.208*	0.143*
Axial length, mm	23.87 ± 0.78	23.58 ± 5.27	23.73 ± 1.11	0.606*	0.899*
Central corneal thickness, μm	551.44 ± 22.17	547.63 ± 32.45	539.65 ± 21.30	0.088*	0.374*
Intraocular pressure, mmHg	15.03 ± 3.11	13.73 ± 3.11	13.67 ± 2.50	0.066*	0.927*
Best-corrected visual acuity	0.94 ± 0.13	0.94 ± 0.10	0.62 ± 0.22	<0.001*	<0.001*
**Colour vision test, n (%)**					
Normal	29 (96.7)	25 (83.3)	15 (50.0)	<0.001^†^	0.006^†^
B-Y defect	0 (0)	2 (6.7)	2 (6.7)		
R-G defect	1 (3.3)	2 (6.7)	2 (6.7)		
Combined	0 (0)	1 (3.3)	11 (36.7)		
Disc-fovea angle (°)	6.67 ± 3.58	7.35 ± 4.17	7.17 ± 3.98	0.781^*^	0.924^*^
Vertical optic disc rotation (°)	1.47 ± 2.18	1.65 ± 2.77	1.58 ± 2.35	0.958^*^	0.987^*^
Horizontal optic disc rotation (°)	1.56 ± 2.23	1.71 ± 3.01	1.69 ± 2.98	0.897^*^	0.992^*^

Data are presented as mean±standard deviation.RNFL, retinal nerve fibre layer; B-Y defect, blue–yellow defect; R-G defect, red–green defect.

^*^Comparison two groups by Student *t*-test.

^†^Comparison between the two groups by the chi-square test and Fisher’s exact test.

*P-*value^a^ comparison between controls and glaucoma patients with temporal RNFL defects.

*P-*value^b^ comparison between glaucoma patients with superotemporal and/or inferotemporal RNFL defects and glaucoma patients with temporal RNFL defects.

Table 2 shows the results of visual field testing among subjects. The mean deviation (MD) of standard automated perimetry (SAP) was similar between NTG eyes with inferotemporal or superotemporal RNFL defects and those with temporal RNFL defects (*P* = 0.992). The visual field index (VFI) values were significantly different between NTG eyes with inferotemporal or superotemporal RNFL defects and those with temporal RNFL defects. Eyes with temporal RNFL defects showed lower VFI values (*P* = 0.041). Short-wavelength automated perimetry (SWAP) and frequency doubling technology (FDT) were significantly different regarding MD between the groups. Eyes with temporal RNFL defects showed lower MD values (*P* = 0.005).

**Table 2:** Comparison of the visual field test of glaucoma patients with temporal RNFL defects with those of glaucoma patients with inferotemporal or superotemporal RNFL defects.

	Control (n = 30)	Glaucoma with supero/inferotemporal RNFL defect (n = 30)	Glaucoma with TemporalRNFL defect (n = 30)	P-value^a^	P-value^b^
**Standard automated perimetry**					
Mean deviation of perimetry, dB	0.02 ± 1.11	−3.07 ± 0.35	−3.07 ± 3.69	<0.001*	0.992*
Pattern standard deviation of perimetry, dB	1.36 ± 0.56	3.82 ± 1.89	3.07 ± 2.32	0.001*	0.058*
VFI, %	99.73 ± 0.45	94.50 ± 1.73	91.96 ± 6.40	<0.001*	0.041*
Pattern of defect, central or cecocentral, n (%)	0 (0)	6 (20)	14 (46.7)	<0.001^†^	0.028^†^
**Short-wavelength automated perimetry**					
Mean deviation of perimetry, dB		−9.15 ± 6.49	−11.15 ± 7.62		0.005*
Pattern standard deviation of perimetry, dB		3.51 ± 2.14	3.46 ± 2.45		0.078*
Pattern of defect, central or cecocentral, n (%)		7 (23.3)	19 (66.7)		0.002^†^
**Frequency-doubling technology perimetry**					
Mean deviation of perimetry, dB		−8.03 ± 5.33	−10.28 ± 7.31		0.005*
Pattern standard deviation of perimetry, dB		4.34 ± 1.25	4.19 ± 1.34		0.061*
Pattern of defect, central or cecocentral, n (%)		4 (13.3)	7 (23.3)		0.253^†^

Data are presented as mean±standard deviation.

VFI, visual field index.

^*^Comparison between the two groups by Student *t*-test.

^†^Comparison between the two groups by chi-squared test and Fisher’s exact test.

*P-*value^a^ comparison between controls and glaucoma patients with temporal RNFL defects.

*P-*value^b^ comparison between glaucoma patients with superotemporal and/or inferotemporal RNFL defects and glaucoma patients with temporal RNFL defects.

A central or cecocentral scotoma was significantly more frequent in NTG eyes with temporal RNFL defects as compared with in NTG eyes with inferotemporal or superotemporal RNFL defects. These results were significantly different between the two groups according to SAP (46.7% vs. 20.0%; *P* = 0.028) and SWAP (66.7% vs. 23.3%; *P* = 0.002), but not so for FDT (23.3% vs. 13.3%; *P* = 0.253).

Peripapillary RNFL thicknesses by OCT were only significantly different between the inferotemporal or superotemporal RNFL defects group and the temporal RNFL defects group at the average (*P* = 0.001) and temporal RNFL thicknesses (*P* < 0.001) (Table 3). RNFL thickness was significantly thinner in clock-hour segments 8, 9 and 10 of NTG eyes with temporal RNFL defects than in NTG eyes with inferotemporal or superotemporal RNFL defects (Table 3). Ganglion cell-inner plexiform layer (GCIPL) thicknesses in the temporal RNFL defects group were significantly thinner than those in the inferotemporal or superotemporal RNFL defects group for average, minimum, superior, superonasal, inferonasal, inferior, inferotemporal, and superotemporal thicknesses (*P* < 0.001 except in the inferotemporal sector, which was *P* = 0.005) (Table 3).

**Table 3:** Comparison of the OCT parameters in glaucoma patients with temporal RNFL defects with those of glaucoma patients with inferotemporal or superotemporal RNFL defects.

	Control (n = 30)	Glaucoma with supero/inferotemporal RNFL defect (n = 30)	Glaucoma with temporal RNFL defect (n = 30)	P-value^a^	P-value^b^
**ONH analysis**					
Rim area	1.31 ± 0.78	0.90 ± 0.18	0.94 ± 0.21	<0.001*	0.432*
Disc area	1.82 ± 0.20	1.98 ± 0.54	2.07 ± 0.43	0.006*	0.736*
Average CDR	0.51 ± 0.12	0.70 ± 0.12	0.71 ± 0.10	<0.001*	0.897*
Vertical CDR	0.46 ± 0.12	0.71 ± 0.10	0.67 ± 0.10	<0.001*	0.161*
Cup volume	0.16 ± 0.13	0.42 ± 0.26	0.41 ± 0.32	<0.001*	0.849*
**RNFL thickness, μm**					
Average	98.47 ± 8.36	81.30 ± 10.85	69.67 ± 13.80	<0.001*	0.001*
Superior	124.53 ± 15.15	96.87 ± 16.39	88.47 ± 21.41	<0.001*	0.093*
Inferior	127.17 ± 10.99	91.60 ± 17.39	88.37 ± 28.71	<0.001*	0.925*
Nasal	68.70 ± 9.81	63.03 ± 9.20	63.27 ± 9.98	0.028*	0.600*
Temporal	73.43 ± 10.01	68.80 ± 11.13	39.63 ± 5.89	<0.001*	<0.001*
p12	125.83 ± 20.49	96.17 ± 22.23	95.80 ± 27.74	<0.001*	0.955*
p1	110.93 ± 18.44	92.83 ± 19.80	83.83 ± 22.89	0.002*	0.109*
p2	85.27 ± 17.69	72.50 ± 13.15	65.83 ± 22.15	<0.001*	0.163*
p3	57.53 ± 8.02	57.30 ± 9.08	54.17 ± 13.25	0.239*	0.290*
p4	63.33 ± 10.52	59.13 ± 9.78	55.43 ± 13.21	0.013*	0.223*
p5	98.83 ± 18.62	79.80 ± 12.29	83.07 ± 24.08	0.006*	0.512*
p6	136.70 ± 16.99	97.10 ± 21.56	96.67 ± 33.36	<0.001*	0.953*
p7	145.93 ± 15.31	73.73 ± 14.75	87.17 ± 35.90	<0.001*	0.066*
p8	74.97 ± 12.47	69.33 ± 17.61	42.97 ± 9.86	<0.001*	<0.001*
p9	58.90 ± 8.25	59.77 ± 8.83	38.60 ± 10.92	<0.001*	<0.001*
p10	86.30 ± 12.91	77.60 ± 14.36	51.43 ± 11.04	<0.001*	<0.001*
p11	136.60 ± 21.84	85.30 ± 13.29	90.57 ± 29.33	<0.001*	0.376*
**GCIPL thickness, μm**					
Average	84.73 ± 4.67	74.10 ± 5.16	57.59 ± 7.98	<0.001*	<0.001*
Minimum	83.10 ± 4.51	64.30 ± 10.09	52.12 ± 7.54	<0.001*	<0.001*
Superior	85.47 ± 5.99	76.47 ± 7.20	57.24 ± 8.06	<0.001*	<0.001*
Superonasal	86.30 ± 5.10	80.90 ± 6.27	56.53 ± 8.45	<0.001*	<0.001*
Inferonasal	84.90 ± 5.05	76.63 ± 5.77	56.00 ± 8.30	<0.001*	<0.001*
Inferior	82.67 ± 4.81	69.60 ± 8.89	57.18 ± 8.65	<0.001*	<0.001*
Inferotemporal	85.33 ± 5.21	68.37 ± 9.89	59.94 ± 8.21	<0.001*	0.005*
Superotemporal	83.83 ± 4.39	72.67 ± 8.83	58.35 ± 8.13	<0.001*	<0.001*

Data are presented as mean ± standard deviation.

ONH, optic nerve head; CDR, cup-to-disc ratio; RNFL, retinal nerve fibre layer; GCIPL, ganglion cell–inner plexiform layer.

^*^Comparison between the two groups by Student *t*-test.

*P-*value^a^ comparison between controls and glaucoma patients with temporal RNFL defects.

*P-*value^b^ comparison between glaucoma patients with superotemporal and/or inferotemporal RNFL defects and glaucoma patients with temporal RNFL defects.

Subgroup analysis of NTG eyes with temporal RNFL defects was done according to the presence of colour vision deficiency. BCVA (*P* = 0.020) and GCIPL parameters were significantly reduced in eyes with colour vision deficiency (Table 4). Central or cecocentral scotoma was significantly more frequent in NTG eyes with temporal RNFL defects with colour vision deficiency than in NTG eyes with temporal RNFL defects without colour vision deficiency (Table 5). These results were significantly different between the two groups on SWAP (86.7% vs. 40.0%; *P* = 0.021), but not apparent on SAP (60.0% vs. 33.3%; *P* = 0.143). Central retinal sensitivity was also significantly decreased in NTG eyes with temporal RNFL defects with colour vision deficiency as compared with in NTG eyes with temporal RNFL defects without colour vision deficiency. These results were significantly different between the two groups for SWAP (*P* = 0.048) but not apparent in SAP (*P* = 0.406).

**Table 4:** Comparison of the clinical characteristics and OCT parameters of patients with temporal RNFL defects according to colour vision deficiency.

	With colour vision deficiency (n = 15)	Without colour vision deficiency (n = 15)	P-value
Age, years	54.46 ± 18.03	54.76 ± 9.65	0.957*
Sex, male:female	5:10	4:11	0.493^†^
Spherical equivalent, diopter	−1.40 ± 2.47	−0.33 ± 2.69	0.280*
Axial length, mm	24.05 ± 1.14	23.22 ± 0.49	0.070*
Central corneal thickness, μm	547.14 ± 15.17	534.22 ± 25.51	0.257*
Intraocular pressure, mmHg	13.80 ± 2.48	12.84 ± 1.99	0.278*
Best-corrected visual acuity	0.53 ± 0.21	0.73 ± 0.20	0.020*
**RNFL thickness, μm**			
Average	67.06 ± 11.11	71.61 ± 16.55	0.395^*^
Superior	84.13 ± 17.51	91.53 ± 24.68	0.363^*^
Inferior	84.06 ± 27.99	84.06 ± 27.99	0.473*
Nasal	62.47 ± 8.11	63.46 ± 11.78	0.795*
Temporal	40.66 ± 3.84	38.76 ± 7.85	0.414*
**GCIPL thickness, μm**			
Average	52.12 ± 2.53	64.71 ± 7.76	0.001*
Minimum	47.62 ± 2.72	58.42 ± 7.91	0.003*
Superior	52.25 ± 3.65	63.14 ± 9.13	0.008*
Superonasal	51.12 ± 2.58	63.42 ± 9.25	0.003*
Inferonasal	50.12 ± 2.53	63.57 ± 7.74	<0.001*
Inferior	51.37 ± 2.77	65.14 ± 7.88	<0.001*
Inferotemporal	54.87 ± 3.87	67.14 ± 7.69	0.002*
Superotemporal	53.50 ± 4.10	64.42 ± 8.99	0.008*

Data are presented as mean ± standard deviation.

RNFL, retinal nerve fibre layer; GCIPL, ganglion cell–inner plexiform layer.

^*^Comparison between the two groups by Mann–Whitney U test.

^†^Comparison between the two groups by Fisher’s exact test.

**Table 5:** Comparison of the visual field tests of patients with temporal RNFL defect according to colour vision deficiency.

	With colour vision deficiency (n = 15)	Without colour vision deficiency (n = 15)	P-value
**Standard automated perimetry**			
Mean deviation of perimetry, dB	−3.59 ± 4.64	−2.55 ± 2.99	0.512^*^
Pattern standard deviation of perimetry, dB	3.59 ± 3.43	2.55 ± 1.91	0.362*
VFI, %	92.29 ± 2.68	91.62 ± 1.73	0.463*
Pattern of defect, central or cecocentral, n (%)	9 (60)	5 (33.3)	0.143^†^
Central retinal sensitivity, dB	35.96 ± 2.82	36.36 ± 1.46	0.406*
**Short-wavelength automated perimetry**			
Mean deviation of perimetry, dB	−11.94 ± 4.62	−10.36 ± 5.14	0.721*
Pattern standard deviation of perimetry, dB	3.61 ± 2.21	3.31 ± 2.09	0.556*
Pattern of defect, central or cecocentral, n (%)	13 (86.7)	6 (40.0)	0.021^†^
Central retinal sensitivity, dB	18.73 ± 2.68	22.06 ± 3.84	0.048*

Data are presented as mean±standard deviation.

VFI, visual field index

^*^Comparison between the two groups by Mann–Whitney U test.

^†^Comparison between the two groups by chi-squared test.

## Discussion

High IOP is one of the main risk factors for the progression of glaucoma. However, it is not sufficiently explained to the changes in the optic nerve head and ongoing loss of retinal ganglion cells in patients with normal IOP. Other ocular factors such as central corneal thickness (CCT), disc size, and myopia are known to be associated with the progression of glaucoma^14^. In addition, systemic risk factors such as migraines, low systemic blood pressure, and other circulatory dysfunction are known to be related with NTG^15,16^. We found no significant differences of these factors between NTG patients with temporal RNFL defects and those with inferotemporal or superotemporal RNFL defects. However, the incidences of colour vision deficiency and decreased BCVA were higher in NTG patients with temporal RNFL defects than NTG patients with inferotemporal or superotemporal RNFL defects.

The incidence of colour vision deficiency is high in the glaucoma patients^17–19^. The cause of acquired colour vision deficiency is the defects in the photoreceptors and the optic nerve fibres^20–24^. In our study, more colour vision deficiency and central or cecocentral VF defects were detected in NTG patients with temporal RNFL defects (Table 1 and Table 2). As shown in the representative case in Fig. 2, initial glaucomatous involvement was present at the temporal RNFL and temporal rim of the optic disc. Central or cecocentral scotoma was the characteristic visual field involvement finding, although some patients showed other types of visual field damage such as the nasal step, arcuate defects, or a combined type.

Additionally, central or cecocentral visual field defects were more easily detected by SWAP (66.7%) as compared with SAP (46.7%) in these patients. As shown in the representative case in Fig. 3, visual field defects were more apparent on SWAP than SAP in both eyes. This result may be related to the colour vision deficiencies found in this subgroup of patients with glaucoma. In our study, half of patients with temporal RNFL defects had colour vision deficiency. The fovea is abundant in the red–green cone, and the surrounding perifoveal region is abundant in the blue cones^25^. It is supposed that the large proportion of red–green colour vision deficiency relates to the high incidence of central/cecocentral scotoma and decrease of visual acuity. However, in our study, most of the patients had both the red-green and blue-yellow colour vision deficiency as previously reported^18^.

The visual acuity of patients with temporal RNFL defects tended to be worse than those of the controls and of patients with inferotemporal or superotemporal RNFL defects. In addition, NTG patients with temporal RNFL defects had significantly decreased VFI values as compared with NTG patients with inferotemporal or superotemporal RNFL defects as well as more central cecocentral visual field defects. The VFI is more affected by defects in the central area than defects in the peripheral area in VF examination^26^. The deterioration result from the other functional test, such as pattern electroretinogram, is also associated with the parafoveal VF defects than peripheral nasal step^27^. Patients with an initial presentation at the central visual field and temporal RNFL defects, as in our study, may have more damage than patients with inferotemporal or superotemporal RNFL defects.

The RGC layer in the macula is thinner in glaucoma patients than normal^28^. GCIPL thickness in NTG patients with temporal RNFL defects was significantly less than among NTG patients with inferotemporal or superotemporal RNFL defects (Table 3). The macula is the area with the highest density of RGCs^12^. This area covers less than 2% of the total retina but has more than 30% of the RGCs^29^. Several studies demonstrated that most RGC damages occur in the more vulnerable inferior macular region in glaucoma patients^12,13,28,30^. In our study, the lessvulnerable superior macula region as well as the more vulnerable inferior macula region showed generalized thinning in the temporal RNFL defects group with a cecocentral visual field defect. Previous studies have analysed that superior macular damage tends to combine the visual field defect closer to fixation^13,30^. Some studies have reported that Alzheimer’s disease is related to the optic nerve and the inner layers of the central macular area damages^31–33^. However, we excluded patients with Alzheimer’s disease and other neurodegenerative diseases like cognitive decline and sensorineural hearing loss from this study in order to eliminate potential bias.

Visual acuity and GCIPL thicknesses were decreased significantly more in patients with colour vision deficiency than in patients without colour vision deficiency in the temporal RNFL defects group (Table 4). In addition, the detection rate of central/cecocentral scotoma was significant higher on SWAP than SAP in the temporal RNFL defects group (Table 2 and Table 5). In our study, MD on FDT and SWAP perimetry was significantly different between NTG patient with temporal RNFL defects and NTG patients with supero/inferotemporal RNFL defects, but not on SAP. As previously reported by Garway-Heath *et al*.^34^, our results showed that the detection of central or cecocentral VF defects in NTG patients with temporal RNFL defects on FDT was 23.3%, lower than those on SAP (46.7%) and SWAP (66.7%).

In the analysis of NTG patients, the discovery of temporal RNFL defects and central/paracentral scotoma allows us to suggest a hypothesis on the aetiology of NTG patients with temporal RNFL defects. Representative ophthalmic diseases with temporal RNFL defects are associated with mitochondrial dysfunction. Mitochondrial optic neuropathies have typical characteristics. One of them is central or cecocentral scotoma with the papillomacular nerve fibre bundle damage^35^. Additionally, dyschromatopsia is one of the earliest signs in mitochondrial optic neuropathies^36,37^. Humans have an increased proportion of mitochondria in the prelaminar optic nerve, and the energy consumption in the prelaminar and laminar optic nerve is high. As a result, optic nerve is susceptible to the mitochondrial dysfunction. Several studies have suggested that alteration of mitochondrial function may be related to glaucoma^38–40^. Primary open-angle glaucoma patients have higher incidences of mitochondrial DNA (mtDNA) mutations and lack of complex-I driven ATP synthesis and respiration^41,42^. Jeoung *et al*.^43^ identified a mtDNA variant in NTG patients.

Based on the findings from this study, further investigations should explore the characteristics of NTG patients with temporal RNFL defects through electrophysiological examinations and contrast sensitivity test as well as other VF tests such as SAP 10-2 and FDT 10-2. Also, more profound genetic analysis of glaucoma patients with temporal RNFL defects could provide valuable information for diagnosis, proper management, and probable genetic counselling in patients and their families.

This study has some limitations. First, a relatively small number of patients were analysed. Second, results of this study were drawn from the NTG patients and cannot be generalised for all types of glaucoma patients and there were some overlapping areas of inferior-temporal/superior-temporal RNFL defect in patients with temporal RNFL defects. However, there were no significant differences in vertical and horizontal opic disc rotation between the groups measuring the RNFL thicknesses in each sectors and there was significantly decreased temporal RNFL in patients with temporal RNFL defects compared to patients with supero/inferotemporal RNFL defects. In addition, the overlap area in the boundary of temporal RNFL defect with p10-11 and p7-8 clock-hour based sector may be interpreted as a reason of insignificant differences between the groups in p11 and p7 clock-hours.Third, patients younger than 65 years old with clear media represent selected NTG patients. However, we had no choice but to set the inclusion criteria of patients strictly to reduce errors in VF, especially SWAP.

In conclusion, NTG patients presenting with temporal RNFL defects and central/cecocentral scotoma had different clinical characteristics, such as colour vision deficiencies and decreased visual acuity. These patients had greater reductions in temporal RNFL thickness and a generalized reduction in GCIPL thickness. Therefore, these patients should be monitored more closely for remnant visual function and disease progression.

## Methods

### Participants

This retrospective and cross-sectional study was performed according to the tenets of the Declaration of Helsinki. It was approved by Institutional Review and Ethics Boards of Seoul St. Mary’s Hospital, South Korea (PC19ROSI0058). The need for written informed consent was waived by our Review Board.We retrospectively reviewed the medical records of patients with glaucoma who were seen by a glaucoma specialist (CKP) from January 2009 to August 2014 at the glaucoma clinic of Seoul St. Mary’s Hospital. Patients with temporal RNFL defects were referred to the neuro-ophthalmology clinic to rule out other optic neuropathies that could present as temporal RNFL defects.

All subjects underwent a medical history review and full ophthalmic examinations, including best-corrected visual acuity (BCVA), refraction, slit-lamp biomicroscopy, intraocular pressure (IOP) assessment using Goldmann applanation tonometry, colour vision test, gonioscopy, fundus examination, central corneal thickness (CCT) measurements using ultrasonic pachymetry (SP-3000; Tomey Corp., Nagoya, Japan), HD-OCT (Carl Zeiss Meditec, Inc. Dublin, CA, USA), standard automated perimetry (24-2 Swedish Interactive Threshold Algorithm; SAP; Humphrey Field Analyser II; Carl Zeiss Meditec), and a measurement of axial length using ocular biometry (IOL Master; Carl Zeiss Meditec). Using the fundus photographs, we measured the disc-fovea angle between the disc centre-fovea line and the horizontal line using ImageJ software (available at http://rsb.info.nih.gov/ij).The disc centre-fovea line was defined as line connecting the centre of the disc with the fovea. The disc centre was assessed as the point where the longest diameter and shortest diameter of the optic disc cross^44^. The location of the fovea was determined as the location of the foveal reflex^45^. Additionally, frequency doubling technology (FDT) perimetry using the Matrix FDT Perimeter (Carl Zeiss Meditec) and short-wavelength automated perimetry (SWAP) using the 24-2 SITA program (ver. 4.1) of the Humphrey Field Analyzer II were performed.

**Figure 1:**
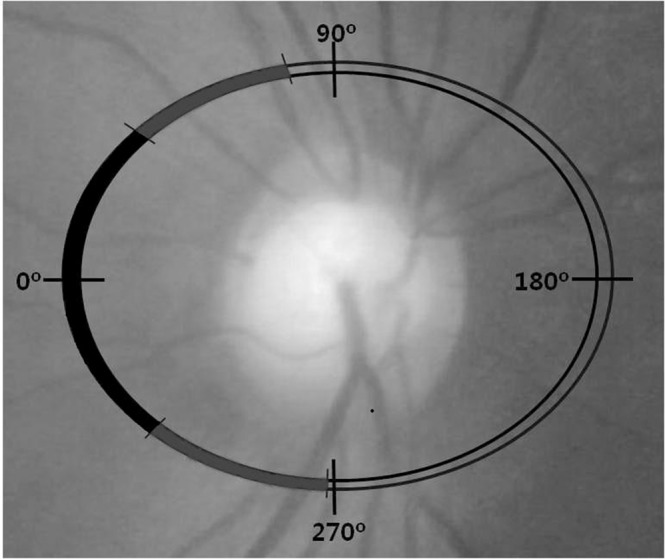
Division of the optic nerve head. Temporal sector of the disc from 311° to 40° (black); superotemporal and inferotemporal sectors of the disc from 41° to 80° and 271° to 310° (gray) in the right eye.

The inclusion criteria for patients with NTG were BCVA of 20/40 or better, spherical refractive error between +3.0 and−6.0 diopters, open anterior chamber angle, and no underlying cause of optic disc damage besides glaucoma. NTG was defined as the presence of an abnormal, glaucomatous optic disc (diffuse or focal thinning of the neuroretinal rim), an abnormal VF consistent with glaucoma, IOP of less than 21 mmHg at least two different times, and an open angle (as determined with gonioscopy) as previously reported^46^. A glaucomatous VF defect was defined as a cluster of three or more points with a probability of less than 5% on the pattern deviation map in at least one hemifield with at least one point with a probability of less than 1%, a VF test result of ‘outside normal limits’ in the glaucoma hemifield test, or a pattern standard deviation (PSD) with a probability of less than 5%^47^. The control group was defined as those with an IOP of less than 21 mmHg with no history of increased IOP, absence of a glaucomatous-appearing disc, no visible RNFL defect according to red-free photography, and normal visual field results^48^. Absence of a glaucomatous disc was defined as an intact neuroretinal rim without peripapillary haemorrhage, notches, or localized pallor^48^. No other ocular diseases were noted during the routine ophthalmological examinations of the control subjects. All individuals included in this study were younger than 65 years with clear optic media. One experienced glaucoma specialist divided the patients with glaucoma into two groups based on the RNFL defect site. The group with temporal RNFL defects included patients with temporal RNFL defects (311–40°) (Fig. 1)^49^. The group with inferotemporal or superotemporal RNFL defects included those with superotemporal (41–80°) or inferotemporal (271–310°) RNFL defects.

**Figure 2:**
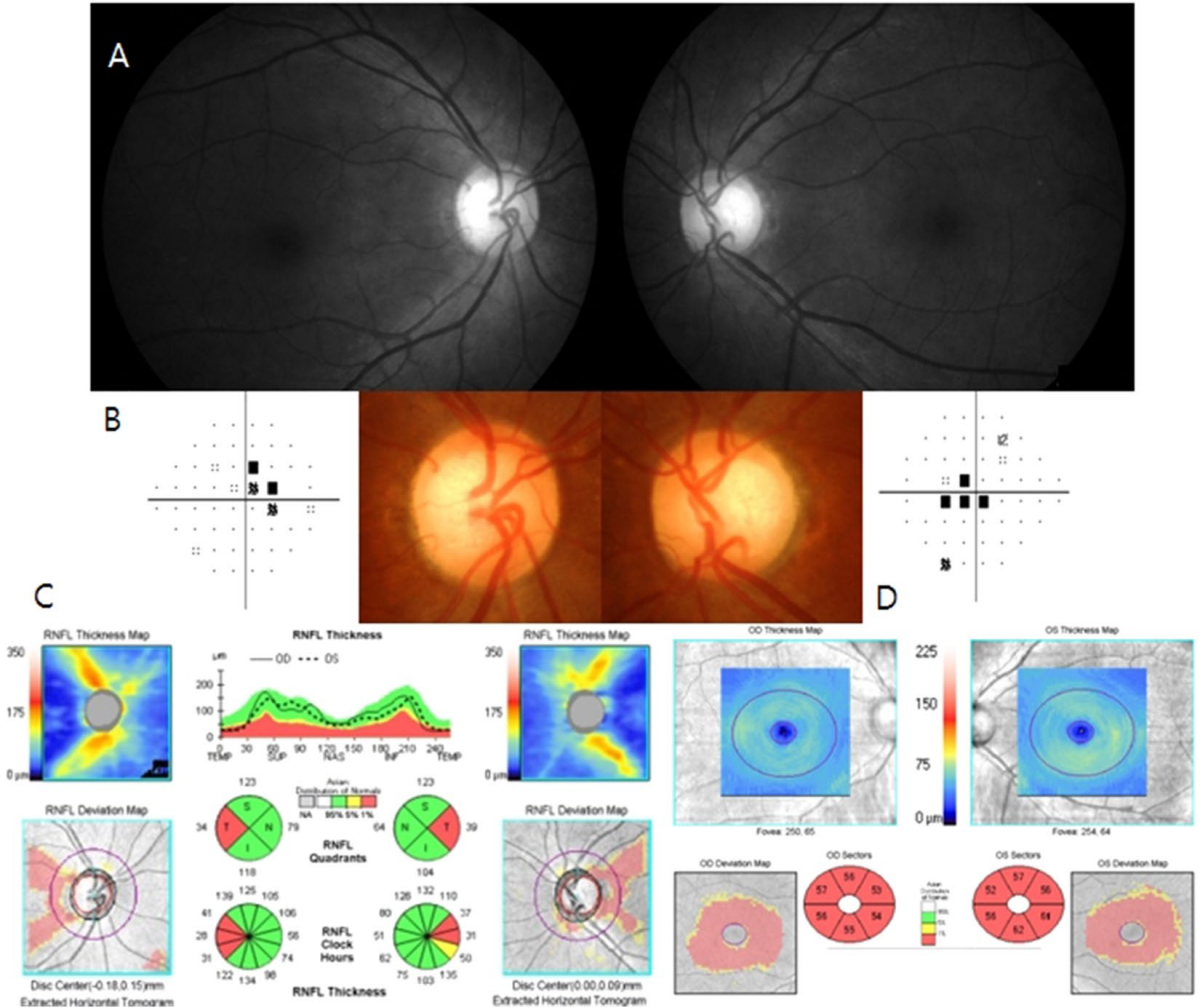
A representative case of eyes with temporal RNFL defects. (**A**) Disc photo shows thinning in the temporal rim of the optic disc and defects in the temporal RNFL area. (**B**) Standard automated perimetry shows central scotomas in both eyes. (**C**) Cirrus OCT optic nerve head images show thinning of the temporal RNFL thicknesses in both eyes. (**D**) Cirrus OCT macular cube scan images show diffuse decreases in GCIPL thickness in both eyes. RNFL, retinal nerve fibre layer; OCT, optical coherence tomography; GCIPL, ganglion cell–inner plexiform layer.

**Figure 3:**
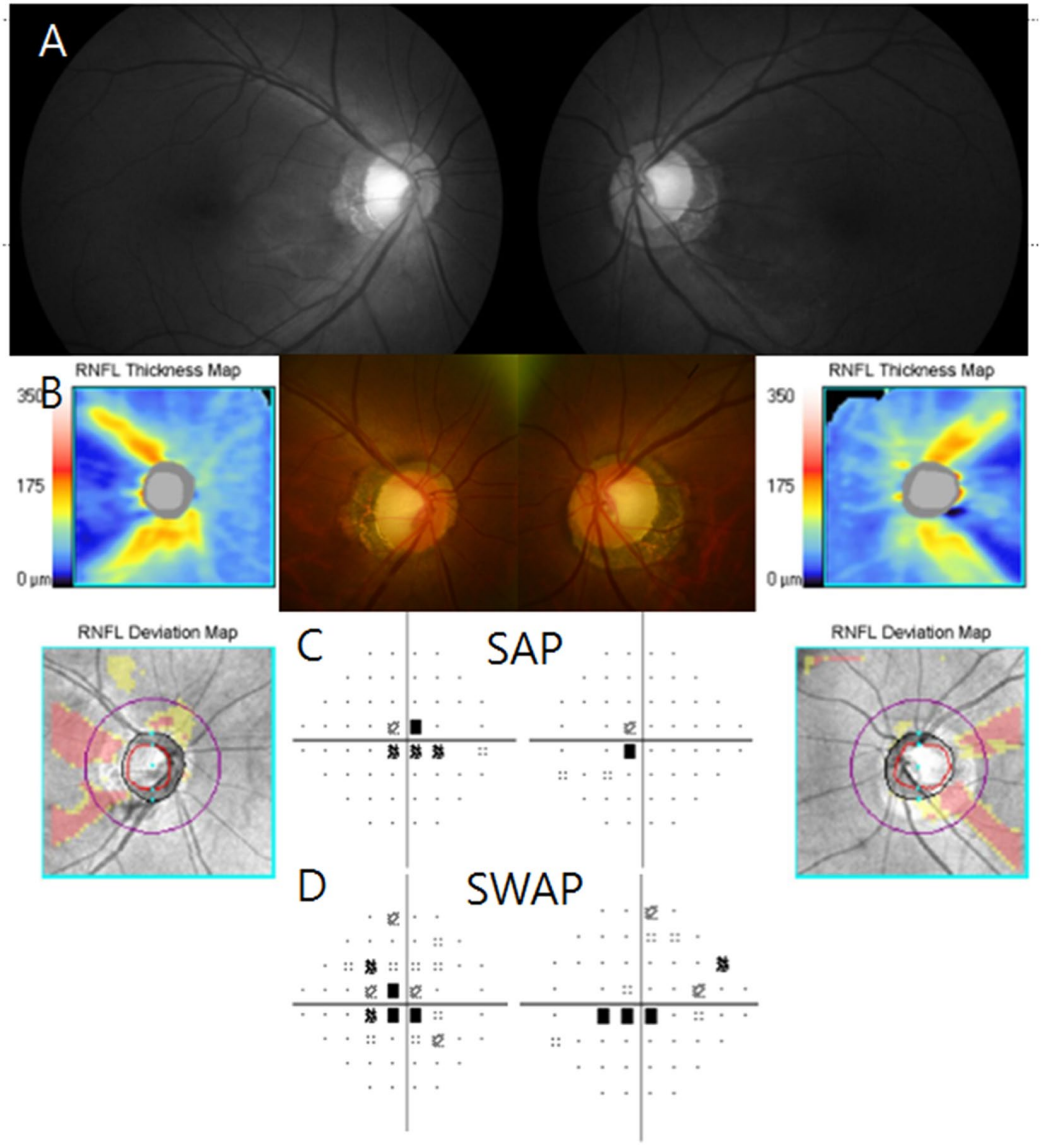
A case of eyes with visual field defect in SAP and SWAP. (**A**) Disc photo shows thinning in the temporal rim of the optic disc and defects in the temporal RNFL area. (**B**) Cirrus OCT optic nerve head images show thinning of the temporal RNFL in both eyes. (**C**) Standard automated perimetry (SAP) shows central scotoma in both eyes. (**D**) Short-wavelength automated perimetry (SWAP) shows central scotoma in both eyes. RNFL, retinal nerve fibre layer; OCT, optical coherence tomography.

To rule out other genetic and compressive optic neuropathies in patients with temporal RNFL defects, we performed brain imaging such as computed tomography or magnetic resonance imaging and mutational analyses including Leber’s hereditary optic neuropathy and dominant optic atrophy. Patients were excluded on the basis of any of the following criteria: a history of any retinal disease; clinical evidence of macular disease; a history of eye trauma or surgery; other optic nerve disease such as ischemic optic neuropathy; and a history of vascular or neurologic disease. Patients with RNFL defects in both temporal and inferotemporal/superotemporal locations or diffuse RNFL defects were excluded. If both eyes were suitable for the study, one eye was chosen for analysis.

### Colour vision test

We performed colour vision tests under standardized lighting conditions, approximately 40 cm away from the patient allowing four seconds to respond to each plate. We used the H-R-R pseudoisochromatic plates (4th edition; Richmond Products Inc, Albuquerque, New Mexico, USA). Each symbol missed with the HRR test was counted as an error and colour vision deficiency was classified in accordance with the test’s instructions^50^.

### Optical coherence tomography

Cirrus HD-OCT instrument software ver. 6.0 was used to analyse the optic disc (optic disc cube 200 × 200 protocol) and macula (macular cube 512 × 128 protocol) for the RNFL and GCIPL measurements^46^. Image was assessed by an experienced examiner blinded to patient’s information. The ONH analysis was conducted to measure rim area, disc area, average cup-to-disc area ratio, vertical cup-to-disc area ratio, and cup volume.The analysis area on the optic disc was centred automatically and the absence of segmentation errors was confirmed for each scan manually. The average RNFL thicknesses in each quadrant (temporal, 311–40°; nasal, 230-121°’ superior, 41–120°; inferior 231–310°) and the clock hour-based sectors(12 sectors of 30°) were obtained for all individuals and analysed. The boundary between superior and temporal quadrant in quadrant map is located between 10 and 11 o’clock in clock-hour map and boundary between inferior and temporal quadrant in quadrant map is located between 7 and 8 o’clock in clock-hour map. The RNFL thickness was represented as green color code when within normal range, yellow color code when depressed to a level <5%, and red color code when depressed to a level <1% according to the OCT built-in normative database classification system^51^. It was considered as RNFL defect when the area was presented as yellow or red color in this study. The GCA algorithm identified the layer from the outer boundary of the RNFL to the outer boundary of the inner plexiform layer^52^. The average, minimum, and sectoral (superotemporal, superior, superonasal, inferonasal, inferior, and inferotemporal) thicknesses of the GCIPL were measured in an elliptical annulus (dimensions, vertical inner and outer radius of 0.5 and 2.0 mm, horizontal inner and outer radius of 0.6 and 2.4 mm, respectively)^52^. Details of the manner in which GCIPL thickness measurements are conducted have been reported previously^53,54^. The rotations of the optic disc around vertical and horizontal optic disc axis were measured as the angle between Bruch’s membrane opening reference plane and horizontal line on OCT scans through the optic disc^55^. The rotation angle was positive if the nasal or superior optic disc border was elevated and the temporal or inferior optic disc border was depressed.

### Visual field testing

Before test, refractive errors of the patients were corrected according to the manufacturer’s recommendations for the SAP, FDT, and SWAP examinations. VFs were considered reliable when the fixation losses were 20% or less and the false-positive and false-negative errors were 15% or less. A field defect was defined as having three or more significant (*P* < 0.05) non-edge-contiguous points with at least one at the *P* < 0.01 level on the same side of the horizontal meridian in the pattern deviation plot as reported previously^56^ and confirmed with two consecutive examinations by the same type of perimetry. The pattern of visual field defects was determined by the location of the visual field defect. Eyes with a defect located within the central 10° of fixation, with or without abnormality outside the central 10°, were defined as having a central or cecocentral scotoma^57^. Retinal sensitivity at the central 10° of fixation was averaged in both SAP and SWAP results. All patients underwent SAP initially. Other VF tests were performed in any sequence within 3 months.

### Statistical analysis

Age, spherical equivalent, axial length, central corneal thickness, IOP, MD, PSD, VFI, ONH parameters, RNFL thicknesses, and GCIPL thicknesses were compared between groups using Student *t*-test or Mann–Whitney U test. The sex ratio, family history, systemic medication history, and pattern of visual field defect were compared using the chi-squared test or Fisher’s exact test. SPSS ver. 19.0 (IBM Corp., Armonk, NY, USA) was used for all statistical analyses. *P*-values of less than 0.05 were considered to be statistically significant.
